# Impact of proficiency in the transcatheter aortic valve implantation procedure on clinical outcomes: a single center retrospective study

**DOI:** 10.1186/s12871-024-02594-7

**Published:** 2024-06-21

**Authors:** Hirotsugu Miyoshi, Satoshi Kamiya, Tsuyoshi Ikeda, Soshi Narasaki, Takashi Kondo, Daiki Syourin, Ayako Sumii, Kenshiro Kido, Sachiko Otsuki, Takahiro Kato, Ryuji Nakamura, Yasuo M. Tsutsumi

**Affiliations:** Department of Anesthesiology and Critical Care, Hiroshima University, 1-2-3 Kasumi, Minami-Ku, Hiroshima, 734-8551 Japan

**Keywords:** Transcatheter aortic valve implantation, General anesthesia, Local anesthesia, Learning curve

## Abstract

**Background:**

We used transcatheter aortic valve implantation (TAVI) procedure time to investigate the association between surgical team maturity and outcome.

**Methods:**

Among patients who underwent TAVI between October 2015 and November 2019, those who had Sapien™ implanted with the transfemoral artery approach were included in the analysis. We used TAVI procedure time and surgery number to draw a learning curve. Then, we divided the patients into two groups before and after the number of cases where the sigmoid curve reaches a plateau. We compared the two groups regarding the surveyed factors and investigated the correlation between the TAVI procedure time and survey factors.

**Results:**

Ninety-nine of 149 patients were analysed. The sigmoid curve had an inflection point in 23.2 cases and reached a plateau in 43.0 cases. Patients in the Late group had a shorter operating time, less contrast media, less radiation exposure, and less myocardial escape enzymes than the Early group. Surgical procedure time showed the strongest correlation with the surgical case number.

**Conclusion:**

The number of cases required for surgeon proficiency for isolated Sapien™ valve implantation was 43. This number may serve as a guideline for switching the anesthesia management of TAVI from general to local anesthesia.

## Introduction

Transcatheter aortic valve implantation (TAVI) is commonly applied worldwide as a minimally invasive treatment for severe aortic stenosis (AS) [1, 2]. TAVI has been reported to improve the long-term life prognosis of AS patients, and the indications for TAVI are expected to expand to asymptomatic and moderate AS patients [3]. As such, the number of patients eligible for TAVI treatment is expected to increase in the future. Anesthesia management for TAVI includes general anesthesia and local anesthesia combined with sedation, and each method has its advantages and disadvantages. Compared with anesthesia management using general anesthesia, anesthesia management using local anesthesia reduces the amount of catecholamines during surgery, stabilizes hemodynamics, promotes postoperative recovery, and leads to reduced medical costs [4]. Furthermore, one of the merits of TAVI anesthesia management with local anesthesia is the shortening of the operation time [5–7]. Therefore, intraoperative management with local anesthesia is selected in hospitals with a large number of TAVI cases. Conversely, intraoperative management of TAVI with local anesthesia has several disadvantages, such as the need for expert skill in respiratory management during sedation, restriction of the use of transesophageal echocardiography (TEE), and difficulty in response to sudden changes [8–12]. It has been reported that the 30-day mortality rate of TAVI patients is lower under local anesthesia, but as this was a report from a large facility, it is necessary to consider the bias of experience [7, 12, 13]. There is controversy regarding whether general or local anesthesia should be used for anesthesia management during TAVI. Therefore, the method of anesthesia management depends on the characteristics of the TAVI team.

As a precondition for transitioning intraoperative management of TAVI from general anesthesia management to local anesthesia management, surgeons must be sufficiently skilled in the relevant surgical techniques. As in many other surgeries, there is an association between the number of surgical experiences and outcomes, with the outcomes of TAVI expected to improve with an increase in the number of surgical experiences. In fact, it has been reported that complications were associated with the number of cases experienced by the operator in TAVI patients [7, 13]. For this reason, it is essential for the operator to be sufficiently accustomed to the surgical technique to ensure safety. Various studies have been conducted to date as an indicator of operator proficiency in TAVI, and the number of experienced cases required to reduce postoperative complications and improve postoperative prognosis has been reported [14–16]. However, many of these reports set postoperative prognosis as the outcome, meaning that many perioperative factors are included in the study results. In other words, the maturity of the surgeon's technique itself was not examined. Therefore, we conducted this study focusing on TAVI techniques to assess the maturity of surgeons in TAVI.

Herein, we focused on the time required for the procedure to place the TAVI valve and the number of experiences and clarified the number of cases required to shorten the time required for the surgical procedure. In addition, we investigated the effect of the number of experiences required for the proficiency of the TAVI procedure calculated by us on the postoperative outcome.

## Methods

### Ethical considerations

This was a single-center retrospective cohort study conducted at Hiroshima University Hospital in Japan. The study was conducted in accordance with the Declaration of Helsinki and was approved by the institutional ethics board of Hiroshima University Hospital (No. E-1463). The need for informed consent was waived by the Ethical Committee for Epidemiology of Hiroshima University due to the study's retrospective nature.

### Study subjects

**Fig. 1:**
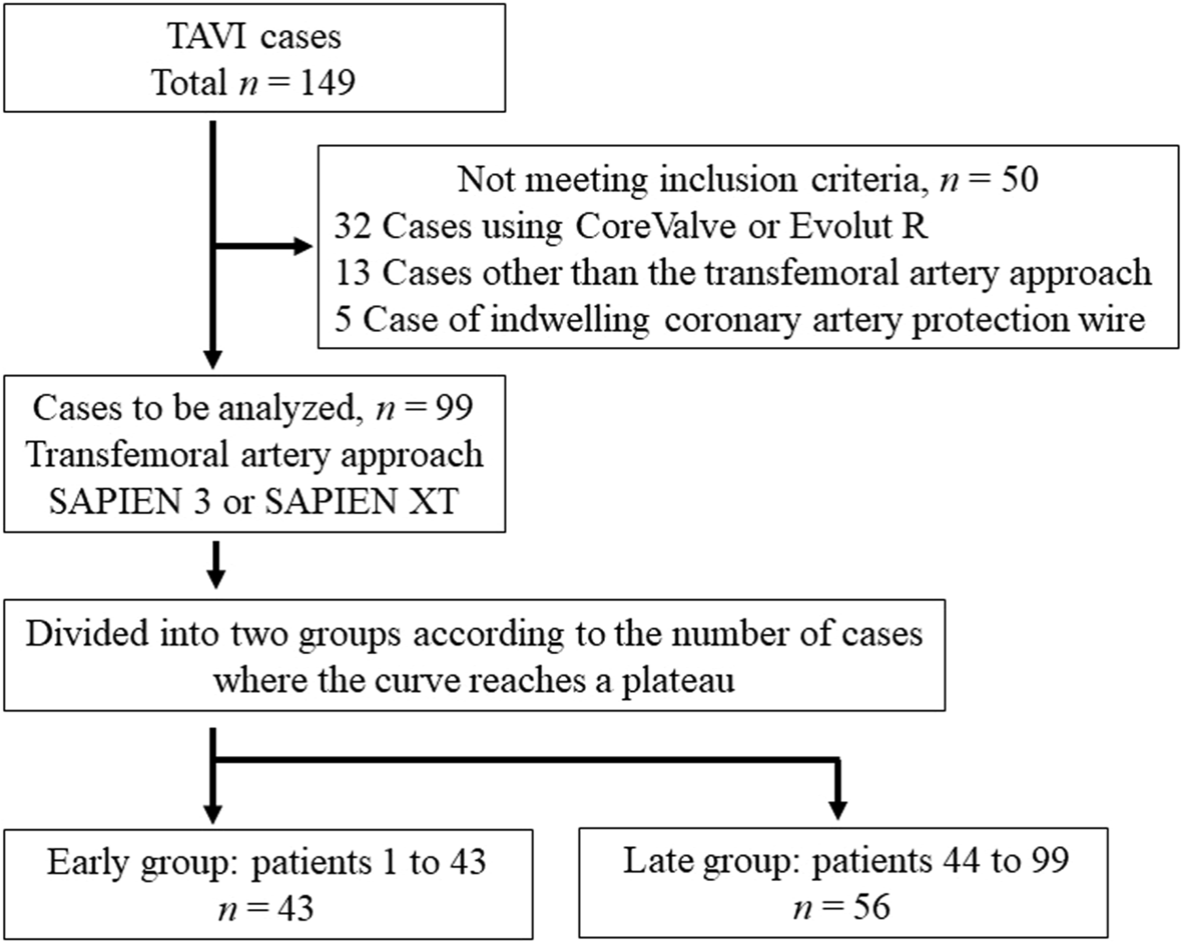
Flow chart showing patient selection criteria and group division. We analyzed 99 out of 149 treated patients. Patients were divided into the Early and Late groups based on the number of cases when the sigmoid curve plateaued. TAVI: transcatheter aortic valve implantation, n: number of patients

We included all patients who underwent TAVI between October 2015 and November 2019 at Hiroshima University Hospital. We focused on patients with a Sapien™ valve (Sapien XT or Sapien 3) (S3, S-XT; Edwards Lifesciences, Inc., Irvine, CA, USA) and transfemoral artery (TF) approach and excluded other patients. Thus, patients who used Evolute™ (CoreValve valve-Medtronic, Minneapolis, MN, USA) or who chose a non-transfemoral approach were excluded from the analysis. In addition, during valve placement, patients who underwent additional procedures other than TAVI valve placement, such as coronary artery protection and percutaneous cardiopulmonary support (PCPS), were excluded. A flowchart of the patient selection process is shown in Fig. 1.

### TAVI team and environment

During the time period of this study, a single TAVI team existed consisting of interventional cardiologists, cardiovascular surgeons, clinical engineers, anesthetists, and nurses. The cardiologists were primarily responsible for catheter placement procedures, and the main operator remained consistent throughout the study period. Midway through the study, one cardiac surgeon was replaced by another experienced surgeon. The anesthesia team consisted of two anesthesiologists, specializing in cardiothoracic anesthesia. The first eight cases were screened on-site initially, and 25 cases were done under the supervision of a proctor. All cases were done with a balloon aortic angioplasty.

### Definition of TAVI procedure time and anesthesia management method

**Fig. 2:**
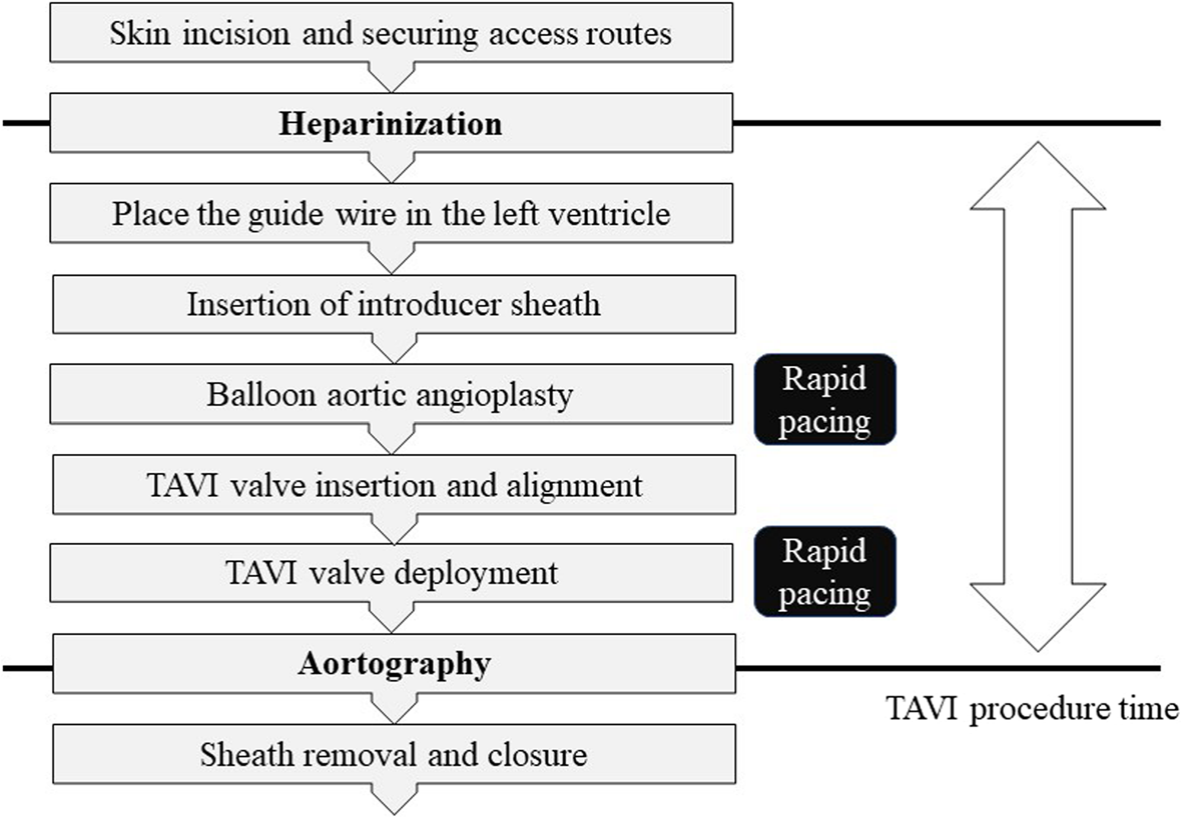
Flow of the TAVI procedure and definition of the TAVI procedure time. The procedure flow of the TAVI is shown. We defined the TAVI procedure time as the time from the heparinization to the confirmation of completed valve placement by aortography. TAVI: transcatheter aortic valve implantation

The TAVI procedure time was defined as the time from the heparinization to the confirmation of completed valve placement by aortography to focus only on the proficiency of the valve placement procedure. Figure 2 shows the flow of the TAVI procedure and the definition of the TAVI procedure time.

Anesthesia management method: All TAVI cases were performed under general anesthesia. Tracheal intubation was performed in all cases, and monitoring was performed using a TEE and a pulmonary artery catheter. After surgery, all patients were extubated in the operating room and subsequently admitted to the intensive care unit (ICU).

### Surveyed factors

We collected the following information as Surveyed factors: Type of implanted Sapien™ valve (Sapien XT or Sapien 3, others: Evolute™), patient background (age, sex, height, weight, body mass index (BMI), and preoperative risk factors (aortic valve orifice area, aortic valve mean pressure gradient, left ventricular ejection fraction (LVEF), frailty score, Society of Thoracic Surgeons (STS) score, Logistic EuroScore, Clinical Frailty Scale score), intraoperative factors (operating time, operating room stay time, TAVI procedure time, radiation exposure dose, contrast agent usage), postoperative course (ICU stay days, postoperative hospital stay, 30-day survival rate), blood test findings (anemia: Hb, renal function: blood urea nitrogen (BUN), creatinine (Cre), myocardial deviation enzymes: troponin, troponin I, troponin T, creatine kinase (CK), and creatine phosphokinase-MB (CK-MB)).

### Statistical analyses

#### Fitting a sigmoidal curve and patient grouping

First, we assigned case numbers in order of experience and plotted the number of experienced patients and valve placement time. At this time, Evolute™ cases were only counted by the number of cases, and the valve placement time was not used in the analysis.

Next, the patients to be analyzed were numbered in order of experience, and the learning curve was fitted with a sigmoid curve to the plot of the valve placement procedure time. Next, the number of cases required for maturation was defined as the number of cases at which the curve reached a plateau. Finally, we used the least-squares method to fit a sigmoidal function to the discrete data obtained for TAVI procedure time and the number of cases.

Furthermore, we divided the patients into the Early and Late groups, defined as patients treated before and after reaching the plateau of the sigmoid curve and compared the two groups in terms of patient background and surveyed factors. We used the Mann–Whitney U test and Chi-square test for statistical comparison of both groups, and p < 0.05 was considered statistically significant.

#### Correlation with surgical procedure time

We investigated the correlation between the TAVI procedure time and survey factors for the patients. Pearson correlation coefficient was used for statistical analysis; values below -0.4 or above 0.4 were considered to be correlated.

## Results

Of the 149 patients treated with TAVI during the study period, 99 were included in the analysis (Fig. 1). Fifty patients were excluded from the analysis because of non-Sapien valve placement, non-TF approach, or additional procedures other than TAVI valve placement. (Detail: Evolute™ 32 patients, trans subclavian approach 4 patients, trans apical approach 9 patients, coronary artery protection 5 patients).

**Fig. 3:**
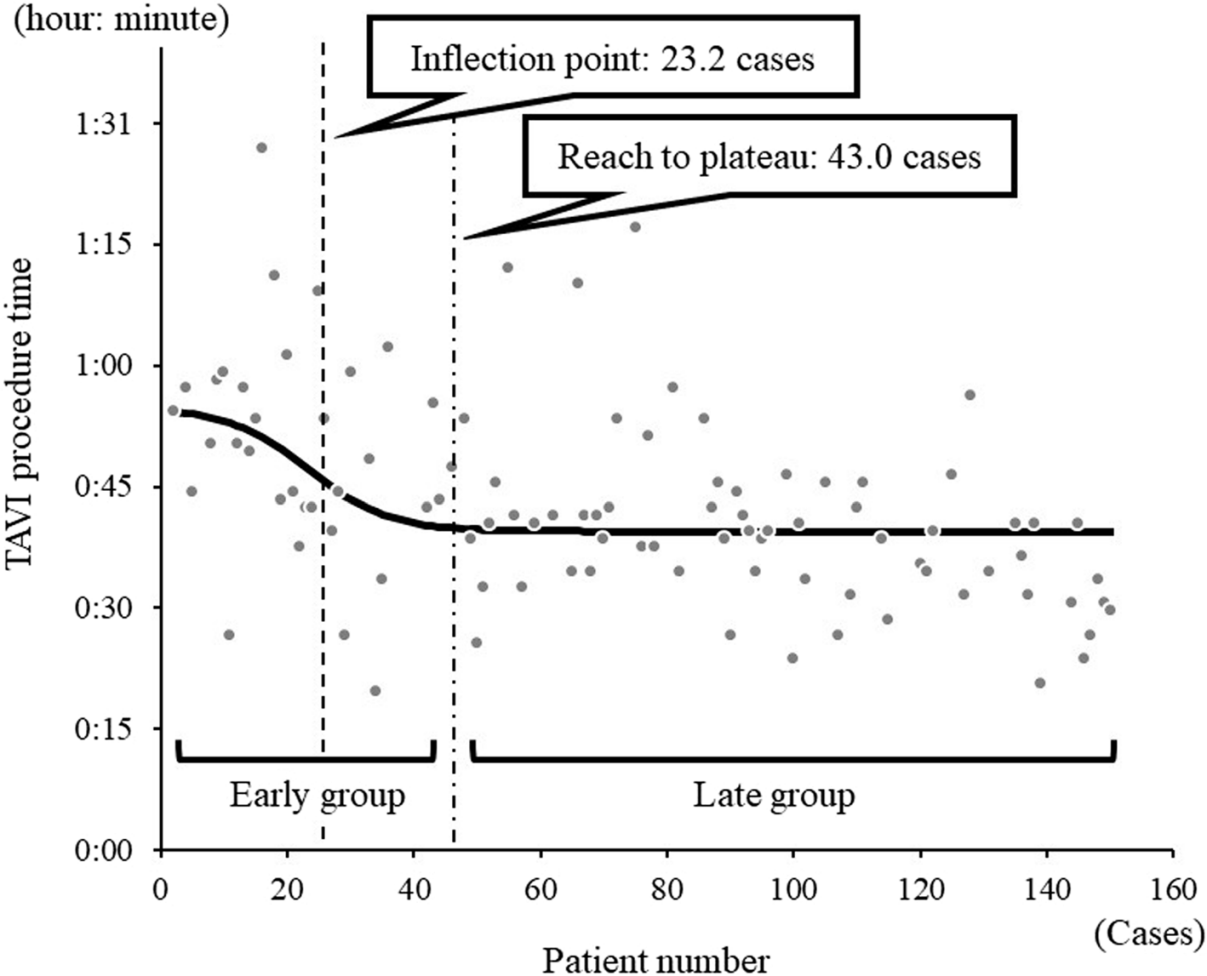
Scatter plot and sigmoid curve of the TAVI procedure time. Scatter plot and sigmoid curve, each data point represents a person undergoing a TAVI procedure meeting inclusion criteria. The y axis is TAVI procedure time. The x axis represents the ordinal number (first, second, third, etc.) of the people meeting inclusion criteria. The first person undergoing a TAVI procedure would be assigned the number 1. The second person would be assigned the number 2 and so on. Only those TAVI procedures meeting inclusion criteria would be plotted on the scatter plot. The scatter plot only shows 33 data points in the early group not 43 data points. The vertical lines showing the inflection point and the plateau need to be adjusted. The inflection point should be at the 23rd data point not at patient number 23. The vertical line separating the early group from the late group should be at the 43 data point (occurs at 58 patient number)

The coefficient of determination of the sigmoid curve was 0.15 (P < 0.0001). The sigmoid curve had an inflection point in 23.2 cases and reached a plateau in 43.0 cases (Fig. 3). From this, it was considered that 43 cases would serve as a measure of the maturity of the surgical technique of TAVI.

We subsequently divided the patients into two groups, the Early and Late groups, defined as patients treated before and after these 43 cases, respectively, and subsequently compared the outcomes of the two groups.

Table 1 shows a comparison of patient backgrounds and study factors between groups. In terms of patient background, aortic valve area and mean aortic valve pressure gradient were significantly lower in the Early group. Contrast agent usage, fluoroscopy time, and radiation exposure were significantly higher in the Early group. Furthermore, myocardial escape enzymes on post operative day 0 (POD 0) were also significantly higher in the Early group.

**Table 1:** Comparison of patient background and surveyed factors between early and late groups

	All patients	Early group	Later group	*p* value
Number of patients (n)	99	43	56	
Age (years)	84 [65–96]	85 [71–92]	83.5 [65–96]	0.062
Gender (male: female)	19: 80	10: 33	9: 47	0.810
Height (cm)	148 [130–174]	148 [134–174]	148 [130–170]	0.633
body weight (kg)	50 [33–80]	48 [33–80]	51 [35–75]	0.375
BMI (kg/m^2^)	23 [16–31]	23 [16–31]	23 [16–31]	0.651
Aortic valve area (cm^2^)	0.7 [0.3–1.1]	0.6 [0.4–1.0]	0.7 [0.3–1.1]	0.007
Mean aortic valve pressure gradient (mmHg)	48 [16–119]	52 [30–119]	45 [16–96]	0.014
Left ventricular ejection fraction (%)	65 [27–75]	65 [27–75]	65 [30–73]	0.840
Hemoglobin (g/dL)	11.5 [6.6–13.9]	11.5 [8.6–13.2]	11.5 [6.6–13.9]	0.651
Frailty score	3 [1–7]	3 [2–6]	4 [1–7]	0.207
STS score	4.2 [1.6–34.2]	4.3 [1.6–34.2]	4.2 [2.3–13.8]	0.782
Euro score	3.5 [1.6–22]	3.5 [1.6–22]	3.3 [1.6–12.4]	0.852
Surgical procedure time (min)	42 [20–88]	46 [20–88]	39 [21–78]	< 0.001
Contrast agent usage (mL)	116 [52–370]	135 [60–275]	110 [52–370]	0.001
X-ray fluoroscopy time (sec)	1320 [654–3174]	1695 [1020–3174]	1141 [654–2280]	< 0.001
Radiation exposure dose (mGy)	378 [19–2194]	524 [31–2194]	320 [19–1404]	< 0.001
Total rapid pacing time (sec)	72 [34–141]	72 [34–141]	72 [50–137]	0.752
BAV rapid pacing time (sec)	17 [17–53]	17 [13–51]	17 [9–53]	0.223
TAVI rapid pacing time (sec)	36 [24–80]	36 [24–63]	35 [25–80]	0.846
Surgery time (min)	92 [61–213]	121 [77–213]	84 [61–166]	< 0.001
Length of stay in operating room (min)	197 [152–327]	209 [159–327]	190 [152–274]	< 0.001
Length of stay in ICU (day)	2 [2–17]	2 [2–17]	2 [2–6]	0.804
Post operative length of hospital stay (day)	10 [5–65]	9 [6–60]	10 [5–65]	0.921
30-day survival (n, %)	99 (100%)	43 (100%)	56 (100%)	-
Cardiac troponin I (ng/mL)				
POD 0	0.5 [0.0–3.1]	0.6 [0.1–3.1]	0.4 [0–2.3]	0.011
POD 1	0.3 [0.1–2.3]	0.3 [0.1–1.7]	0.3 [0.1–2.3]	0.456
Cardiac troponin T (ng/mL)				
POD 0	0.3 [0–2.4]	0.3 [0–2.4]	0.2 [0–1]	0.019
POD 1	0.2 [0–0.8]	0.2 [0.1–0.8]	0.2 [0–0.6]	0.085
Creatine kinase (IU/L)				
Preoperative	66 [11–1338]	65 [18–1338]	66 [11–225]	0.893
POD 0	111 [14–871]	114 [62–871]	110 [14–238]	0.154
POD 1	90 [11–562]	90 [25–562]	93 [11–333]	0.819
CK-MB (IU/L)				
Preoperative	11 [4–19]	11 [4–19]	12 [8–14]	0.632
POD 0	20 [7–50]	20 [7–50]	20 [8–44]	0.284
POD 1	11 [3–32]	9 [5–32]	11 [3–31]	0.401
Blood urea nitrogen (mg/dL)				
POD 0	17 [7–96]	17 [7–96]	15 [7–56]	0.179
POD 1	13.7 [6–80]	14 [6–80]	13 [7–58]	0.364
Creatinine (mg/dL)				
POD 0	0.8 [0.4–4.6]	0.8 [0.4–4.6]	0.8 [0.5–3.1]	0.235
POD 1	0.7 [0.4–4.8]	0.7 [0.4–4.8]	0.7 [0.4–3.7]	0.867

A comparison of patient background factors and surveyed factors between the Early and Late groups. Values are shown as median [min–max range] or number. A Mann–Whitney U test was used for Patients background except Gender, and a chi-square test was used for Gender. A *p*-value < 0.05 was considered to indicate statistical significance

Table 2 shows the correlation coefficients between the TAVI procedure time and survey factors. Fluoroscopy time and surgical case number significantly correlated with TAVI procedure time. In addition, the amount of contrast medium used, the amount of radiation exposure, and the difference in mean aortic valve pressure gradient showed a slight correlation with the TAVI procedure time.

**Table 2:** Correlation coefficient between survey factors and TAVI procedure time

Patient number	-0.47
Age (years)	0.09
Gender (male: female)	0.00
Height (cm)	-0.08
body weight (kg)	0.00
BMI (kg/m^2^)	0.05
Aortic valve area (cm^2^)	-0.18
Mean aortic valve pressure gradient (mmHg)	0.25
Ejection fraction (%)	-0.13
Frailty score	-0.15
STS score	-0.13
Euro score	-0.04
Contrast agent usage (mL)	0.24
X-ray fluoroscopy time (sec)	0.60
Radiation exposure dose (Gy)	0.31
Rapid pacing total time (sec)	0.18
Length of stay in ICU (day)	-0.05
Post operative length of hospital stay (day)	-0.03

Correlations between TAVI procedure time and surveyed factors are shown. The Pearson correlation coefficient was used for statistical analysis; values below -0.4 or above 0.4 were considered correlated

## Discussion

Of a total of 149 patients who underwent TAVI under general anesthesia at a single hospital, we analyzed 99 who underwent Sapien™ valve placement via the TF approach. Focusing on the technique of TAVI valve placement, we analyzed the time required for the surgical procedure and the degree of maturity of the surgical procedure. When we plotted experience cases and surgical procedure time and fitted them to a sigmoid curve, the number of cases in which the surgical procedure time reached a plateau was 43. Further analysis revealed that patients in the Late group had a shorter operating time, shorter stay in the operating room, less contrast media, less radiation exposure, and less myocardial escape enzymes than the Early group. Furthermore, the surgical procedure time is strongly correlated with a surgical case number.

Several studies have been conducted to investigate the proficiency of TAVI. In a study of 177 patients, Mattia Lunardi and colleagues reported that experience with 54 empirical cases were required to reduce serious complications, and 32 cases were needed to improve 30-day survival [17]. Alli et al. reported that TAVI proficiency in 30 patients was required to reach a plateau of mastery [14]. In a report of 1,752 patients in a national TAVI registry from Japan, the association between the 30-day postoperative complication rate and the number of cases experienced by the surgeon was evaluated, revealing that about 20 cases were needed to reduce the risk of postoperative complications [16] In addition, data from the US registry shows only about 100 patients have a stable postoperative course [18]. An article in the literature utilizes combined data from the Transcatheter Valve Therapy (TVT) registry. It examines the learning curves and the relationship between procedural volume and outcomes for Balloon-expandable valve Implants, published in 2019. The registry encompasses 61,949 valve implants. By amalgamating data from all sites in the registry, the sequence of cases was plotted against 30-day mortality and stroke rate. The learning curve showed a change in slope in case 55, after which the curve leveled off [19]. All of these reports indicate that outcome improves as the TAVI team gains more experience with surgical cases, and it is thought that about 50 cases are necessary for proficiency in TAVI. However, these studies set the endpoints as postoperative complications and a 30-day survival rate and did not investigate the maturity of the surgical technique itself. In other words, postoperative prognosis reflects many factors, including preoperative and postoperative ward management. A variety of factors improve outcomes after TAVI, including improved procedural techniques and dissemination of best practices, extensive knowledge sharing, rigorous supervision, ongoing case support, a heart team approach, and improved device technology [19]. On the other hand, since intraoperative complications significantly impact mortality in TAVI, we believe that an analysis that focuses on the surgical technique is necessary [20–22]. This is because information on postoperative management can be supplemented by obtaining information from clinical reports or guideline, but surgical procedures must be mastered through real-world experience. Therefore, the surgeon's proficiency in surgical technique should be evaluated in isolation from other factors. In fact, in many surgical procedures, the time spent on the procedure is considered the maturity of the surgical technique [23–25]. Based on this, we analyzed the time spent on the surgical procedure to define the proficiency of the TAVI procedure. As a result, we found that the number of cases required for proficiency in the TAVI procedure was at least 43.

Whether anesthesia management for TAVI is performed with general or local anesthesia depends on the characteristics of the TAVI team. TAVI is generally performed predominantly under local anesthesia in Europe and general anesthesia in North America. In addition, local anesthesia tends to be selected at facilities where TAVI is frequently performed. The complication rate has been reported to be lower, and the patient outcome is better in facilities where TAVI is performed more frequently [26]. Anesthesia management with local anesthesia has been reported to shorten operating room stay time, reduce the amount of catecholamine used during surgery, stabilize hemodynamics, improve recovery after surgery, and reduce the medical costs compared to anesthesia management with general anesthesia [4]. In addition, postoperative delirium and respiratory complications tend to be lower with local anesthesia management [27, 28]. Conversely, reported advantages of general anesthesia include less patient discomfort during surgery, reliable immobilization, use of TEE, and reduced paravalvular leakage [8, 9]. The rate of unexpected transition from local anesthesia management to general anesthesia management is 2–5%, and management with general anesthesia is required, especially in sudden changes. [9, 29,-32] Considering these reports, we believe that local anesthesia management is suitable for facilities in which the surgical team is sufficiently skilled in TAVI, while general anesthesia management is suitable for facilities that are immature for TAVI. In our study data, the experience of 43 TAVI cases was considered to be one measure of the maturity of the surgeon's technique. In other words, this number of cases may serve as a guideline for switching the anesthesia management of TAVI from general to local anesthesia.

The number of TAVI cases experienced has been reported to be negatively correlated with the incidence of complications and patient outcomes [26]. Our data further showed a correlation between the number of surgeries and duration of surgical procedure, but the length of surgical procedure did not affect 30-day survival or length of hospital stay. This may have interfered with the effect of operative time, as multiple factors other than team management skills are involved in postoperative outcomes. Conversely, since the amount of contrast medium used is involved in developing postoperative acute kidney injury, long-term follow-up may affect renal function [33]. Regarding myocardial escape enzymes, it has been reported that elevated troponin T levels show a weak association with postoperative left heart dysfunction and that elevated troponin I does not affect postoperative mortality [34, 35]. Elevated myocardial escape enzymes were also observed in our patients in the Early group, but the impact on life prognosis was unclear from our results.

Our study has several limitations. First, we only analyzed patients who underwent an isolated Sapien™ TAVI. Some of the skills used during the 15 excluded patients in the early group overlap with the study group. Second, in our country the first 25 cases will be carried out under the supervision of a proctor. We speculate that both of these facts may cause the number of cases required for a surgeon's proficiency as calculated from the learning curve to be underestimated. Next, the aortic valve opening area of patients tended to be smaller in the Early group than in the Late group. This is thought to be because TAVI was given priority to patients with severe AS when TAVI was started. Therefore, it is possible that the TAVI procedure in the Early group became more difficult. However, since the correlation between the TAVI procedure time and the aortic valve opening area was low, we believe this bias in the patient background has little effect on the results. While these biases might have an effect on the exact number of cases, observation of a plateauing of the reduction in TAVI procedure time remains a valid measure of the maturity of the operator’s skills.

## Conclusion

The number of cases required for surgeon proficiency in TAVI using Sapien™ was 43 cases. Compared to the Early group, patients in the Late group required less contrast medium and received less radiation during surgery, and the fluoroscopy time was shorter. In addition, the postoperative elevation of myocardial escape enzymes was significantly lower in the Late group. This number of cases may serve as a guideline for switching the anesthesia management of TAVI from general anesthesia to local anesthesia.

## Data Availability

TThe datasets used and/or analyzed during the current study are available from the corresponding author on reasonable request.

## Abbreviations

TAVI: Transcatheter aortic valve implantation
AS: Aortic stenosis
TEE: Transesophageal echocardiography
PCPS: Percutaneous cardiopulmonary support
ICU: Intensive care unit
BMI: Body mass index
LVEF: Left ventricular ejection fraction
STS: Society of thoracic surgeons
Hb: Hemoglobin
BUN: Blood urea nitrogen
Cre: Creatinine
CK: Creatine kinase
CK-MB: Creatine phosphokinase-MB
POD: Postoperative day

## References

[1] Nishimura RA, ACC/AHA Task Force Members (2014). AHA/ACC Guideline for the management of patients with valvular heart disease: executive summary: a report of the American college of cardiology/American heart association task force on practice guidelines. Circulation..

[2] Vahanian  A (2012). Joint task force on the management of valvular heart disease of the european society of cardiology (ESC) European association for cardio-thoracic surgery (EACTS). Guidelines on the management of valvular heart disease (version 2012). Eur Heart J.

[3] Leon  MB, PARTNER 2 Investigators (2016). Transcatheter or surgical aortic-valve replacement in intermediate-risk patients. Engl J Med.

[4] Maldonado Y, Baisden J, Villablanca PA, Weiner MM, Ramakrishna H (2018). General Anesthesia Versus Conscious Sedation for Transcatheter Aortic Valve Replacement-An Analysis of Current Outcome Data. J Cardiothorac Vasc Anesth.

[5] Eskandari M, UK TAVI Steering Committee and the National Institute for Cardiovascular Outcome Research (2018). Comparison of general anaesthesia and non-general anaesthesia approach in transfemoral transcatheter aortic valve implantation. Heart.

[6] Droppa M (2019). Comparison of safety and periprocedural complications of transfemoral aortic valve replacement under local anaesthesia: minimalist versus complete Heart Team. EuroIntervention.

[7] Husser  O, GARY Executive Board (2018). Conscious sedation versus general anesthesia in transcatheter aortic valve replacement: the german aortic valve registry. JACC Cardiovasc Interv.

[8] Oguri  A, FRANCE 2 Registry Investigators (2014). Clinical outcomes and safety of transfemoral aortic valve implantation under general versus local anesthesia: subanalysis of the French Aortic National CoreValve and Edwards 2 registry. Circ Cardiovasc Interv.

[9] Maas EH, Pieters BM, Van de Velde M, Rex S (2016). General or Local Anesthesia for TAVI? A Systematic Review of the Literature and Meta-Analysis. Curr Pharm Des.

[10] Brecker  SJ, ADVANCE Study Investigators (2016). Impact of anesthesia type on outcomes of transcatheter aortic valve implantation (from the Multicenter ADVANCE Study). Am J Cardiol.

[11] Hahn RT (2015). Recommendations for comprehensive intraprocedural echocardiographic imaging during TAVR. JACC Cardiovasc Imaging.

[12] Villablanca PA (2018). Comparison of local versus general anesthesia in patients undergoing transcatheter aortic valve replacement: A meta-analysis. Catheter Cardiovasc Interv.

[13] Hyman MC (2017). Conscious Sedation Versus General Anesthesia for Transcatheter Aortic Valve Replacement: Insights from the National Cardiovascular Data Registry Society of Thoracic Surgeons/American College of Cardiology Transcatheter Valve Therapy Registry. Circulation.

[14] Alli OO (2012). Transcatheter aortic valve implantation: assessing the learning curve. JACC Cardiovasc Interv.

[15] Thivilliers AP (2020). The learning curve in transcatheter aortic valve implantation clinical studies: A systematic review. Int J Technol Assess Health Care.

[16] Yamawaki M M (2018). A proctoring system to manage the learning curve associated with the introduction of transcatheter aortic valve implantation in Japan. Heart Vessels.

[17] Lunardi M (2016). Clinical outcomes of transcatheter aortic valve implantation: from learning curve to proficiency. Open Heart.

[18] Carroll JD (2017). Procedural Experience for Transcatheter Aortic Valve Replacement and Relation to Outcomes: The STS/ACC TVT Registry. J Am Coll Cardiol.

[19] Russo MJ (2019). Case Volume and Outcomes After TAVR With Balloon-Expandable Prostheses: Insights From TVT Registry. J Am Coll Cardiol.

[20] Billings FT, Kodali SK, Shanewise JS (2009). Transcatheter aortic valve implantation: anesthetic considerations. Anesth Analg.

[21] Roselli EE, Lytle BW (2014). Emergency use of cardiopulmonary bypass in complicated transcatheter aortic valve replacement: importance of a heart team approach. J Thorac Cardiovasc Surg..

[22] Seiffert  M (2013). Severe intraprocedural complications after transcatheter aortic valve implantation: calling for a heart team approach. Eur J Cardiothorac Surg.

[23] Pernar LIM, Robertson FC, Tavakkoli A, Sheu EG, Brooks DC, Smink DS (2017). An appraisal of the learning curve in robotic general surgery. Surg Endosc.

[24] Sng KK (2013). The multiphasic learning curve for robot-assisted rectal surgery. Surg Endosc.

[25] Bokhari MB, Patel CB, Ramos-Valadez DI, Ragupathi M, Haas EM (2011). Learning curve for robotic-assisted laparoscopic colorectal surgery. Surg Endosc.

[26] Handa  N (2018). Japanese TAVR registry participants. learning curve for transcatheter aortic valve implantation under a controlled introduction system - initial analysis of a Japanese nationwide registry. Circ J.

[27] Abawi M (2016). Incidence, Predictive Factors, and Effect of Delirium After Transcatheter Aortic Valve Replacement. JACC Cardiovasc Interv.

[28] Goren O (2015). Sedation or general anesthesia for patients undergoing transcatheter aortic valve implantation–does it affect outcome? An observational single-center study. J Clin Anesth.

[29] O' Sullivan KE (2014). Is local anesthesia the optimum strategy in retrograde transcatheter aortic valve implantation? A systematic review and meta-analysis. Thorac Cardiovasc Surg.

[30] Fröhlich GM (2014). Local versus general anesthesia for transcatheter aortic valve implantation (TAVR)–systematic review and meta-analysis. BMC Med.

[31] Gauthier C (2015). Mid-term survival after transcatheter aortic valve implantation: Results with respect to the anesthetic management and to the access route (transfemoral versus transapical). Ann Card Anaesth.

[32] Ehret C (2017). Is local anaesthesia a favourable approach for transcatheter aortic valve implantation? A systematic review and meta-analysis comparing local and general anaesthesia. BMJ Open.

[33] Yamamoto M (2013). Renal function-based contrast dosing predicts acute kidney injury following transcatheter aortic valve implantation. JACC Cardiovasc Interv.

[34] Sato T (2020). The determinants and outcomes of myocardial injury after transcatheter aortic-valve implantation: SAPIEN 3 study. Cardiovasc Revasc Med..

[35] Stundl A (2017). Periprocedural Myocardial Injury Depends on Transcatheter Heart Valve Type But Does Not Predict Mortality in Patients After Transcatheter Aortic Valve Replacement. JACC Cardiovasc Interv.

